# Thomas Tursz (1946–2018)

**DOI:** 10.1002/1878-0261.12361

**Published:** 2018-08-02

**Authors:** Ulrik Ringborg, Julio E. Celis

**Affiliations:** ^1^ Cancer Center Karolinska Stockholm Sweden; ^2^ Danish Cancer Society Research Center Copenhagen Denmark

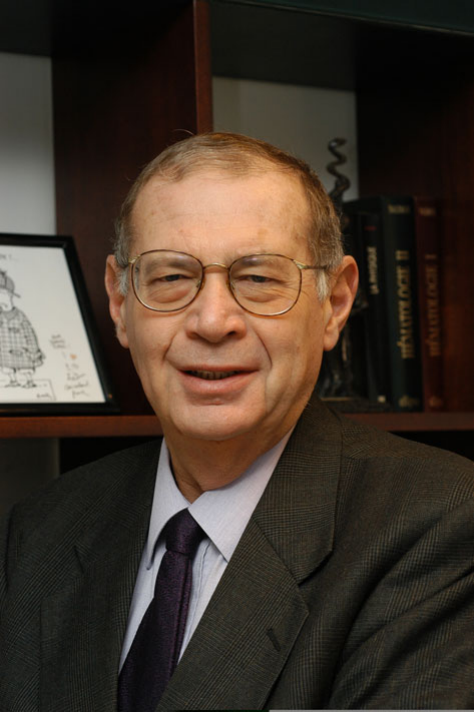

Professor Thomas Tursz, born in Krakow, Poland, in 1946, died on April 27. He was Professor of Oncology at the Faculty of Medicine Paris‐Sud since 1986 and General Director of the Institut Gustave Roussy (1994–2010). Thomas was the leader of the French Doctoral School of Oncology which he founded in 1999, and President of the French Federation of Comprehensive Anticancer Centres (FNCLCC) from 2004 to 2010. He was highly involved in the European Organization for the Research and Treatment of Cancer (EORTC) as both Chairman of the Scientific Advisory Committee (2003–2006) and Vice President of the Board (2006–2009). His experience as President of the FNCLCC was crucial for the Organization of European Cancer Institutes (OECI) when he acted as President from 2002 to 2005.

Thomas acquired basic experience of tumour biology when he was a postdoc in George Klein′s laboratory at the Karolinska Institutet (KI). He had a particular interest in the biology of virus‐induced tumours as well as immunological responses including the role of thioredoxin in lymphocytes infected by Epstein–Barr virus. From 1994 to 1996, he acted as President of the International Association for Research on Epstein–Barr virus and Associated Diseases. In the clinical research area, he conducted a number of important clinical trials in breast cancer, lung cancer and soft‐tissue sarcomas. He had a particular interest in cytokines and gene therapy, and his clinical research activities were further disseminated to the European level when he was the Chairman of the Sarcoma Group of the EORTC (1993–1996).

Thomas received several prestigious awards such as the Prix de Cancérologie from the French National League Against Cancer (1979), the Bernard‐Halpern Immunology Award (1983), the Rosen Oncology Award (1989), the Grand Prix in Oncology from the Academy of Medicine (1992), the Hamilton Fairley Award for clinical research (1998) and the Prix de Rayonnement Français (2001). He was the author of 350 international scientific publications. Thomas was also an esteemed member of the Editorial Board of Molecular Oncology ever since its creation in 2007.

As a close friend, Thomas was a fascinating person. He was well educated in humanistic disciplines with a specific interest in art, literature and politics. His marvellous sense of humour together with his vast experience in both basic biology and clinical oncology made him a visionary with a comprehensive vision for the future of patients with cancer and oncology in general. Already 18 years ago, we had discussions about the need for the strategic development of oncology based on modern cancer biology aimed at translational research. It was quite clear for Thomas that the present organizations involved in cancer care and research must be adapted to modern biology by improving the multidisciplinary structure of cancer care and quality‐assured translational cancer research environments. On the board of the OECI, his argumentation for creating a European methodology for designation of Comprehensive Cancer Centres (CCCs) was convincing, and as a result, a procedure was put in place in 2007.

Thomas was aware of the lack of critical mass in cancer research centres concerning advanced technological resources as well as patients and competencies, and he argued for formal collaborations between cancer research centres. He met, however, with the same problem as Umberto Veronesi when in the 1990s tried to engage 8–10 research‐oriented cancer centres to collaborate; the cancer community was not yet mature enough for such organizational innovations. To address this problem, Thomas became a member of the ‘Working Group on the Coordination of Cancer Research in Europe’ conveyed by the former Commissioner for Research and Innovation Philippe Busquin, whose recommendations led to the funding in FP6 of the EUROCAN +PLUS project. This project strongly supported the development of a European platform for translational cancer research composed of interconnected cancer centres with common projects to accelerate rapid advances in knowledge and their translation into better cancer care and prevention. The project also highlighted the importance of CCCs as they link research and education with therapeutics and prevention, and connect research with the healthcare systems. In fact, when directors of 16 cancer research centres agreed on a declaration to define the Platform concept and its implementation, Thomas suggested naming it the ‘Stockholm Declaration’. His contributions continued in the EurocanPlatform project which delivered the consortium Cancer Core Europe. Today Gustave Roussy, with his successor Alexander Eggermont, is chairing the consortium that has become an engine and a hub to optimize European efforts to fight cancer in partnership.

Thomas played a significant role in moving cancer research and care into new innovative strategies, not only in France but also internationally. At a personal level, a strong friendship developed over the years. His interest in KI went back to his postdoc period at the Tumour Biology department, and he was always a strong supporter of the Cancer Center Karolinska. His contribution to the international evaluation of cancer research at KI in 2000, chaired by Robert Weinberg, was instrumental in persuading the KI leadership to move towards translational cancer medicine. We will remember Thomas as a leading colleague in cancer care and research and as a close friend.

